# Comparative Electrophoretic Analysis Between the Protein Content in Human and Donkey Milk Samples—A Study Covering the Long-Term Lactation Period

**DOI:** 10.3390/foods14173083

**Published:** 2025-09-02

**Authors:** Ana Stoyanova Georgieva, Nikolina Naydenova, Donika Ivanova

**Affiliations:** 1 Department of Animal Husbandry—Ruminants and Animal Product Technologies, Section “Milk and Dairy Products”, Agricultural Faculty, Trakia University, 6000 Stara Zagora, Bulgaria; a.georgieva.stz@gmail.com (A.S.G.); nikolina.zheleva@trakia-uni.bg (N.N.); 2 Department of Pharmacology, Animal Physiology, Biochemistry and Chemistry, Faculty of Veterinary Medicine, Trakia University, 6000 Stara Zagora, Bulgaria; 3 Department of Medicinal Chemistry and Biochemistry, Faculty of Medicine, Trakia University, 6000 Stara Zagora, Bulgaria

**Keywords:** human breast milk, donkey milk, protein fraction, SDS polyacrylamide gel electrophoresis analysis

## Abstract

Human milk contains a wide variety of proteins, possessing antimicrobial and immunomodulatory activities, which are essential for normal infant growth. Over the past few years, the widespread interest in milk’s nutritional quality and its association with infant health care has led to scientific research on the composition of different milk types. In this study, the similarity between protein content in human and donkey milk during the ninth-month lactation period is estimated. Our results demonstrate that donkey milk may be a valid substitute for cow’s milk to supplement the nutrition of allergic children.

## 1. Introduction

It is well known that human milk is the best option for feeding newborns because it is uniquely tailored to meet a child’s needs. It contains a wide variety of proteins, which are easily digestible and provide a balanced source of amino acids for fast-growing infants. Additionally, human milk proteins possess antimicrobial and immunomodulatory activities and are involved in the development of the intestinal mucosa and other organs in newborns [1]. It is well-documented that exclusively breastfeeding for the first six months of life is associated with a decrease in the incidence of infection and chronic diseases, as well as the risk of type 2 diabetes and obesity in later life [2,3,4,5,6]. The World Health Organization (WHO) recommends exclusive breastfeeding for about 6 months, followed by the introduction of complementary foods and continued breastfeeding for 1 year or longer, depending on the mutual desires of the mother and infant [2,3,7]. However, in some cases, health conditions affecting the mother, such as antibiotic therapy or insufficient breast milk, result in only temporary or no breastfeeding.

Various infant formulas have similarities to human breast milk, but no perfect substitute has been found [8]. Additionally, some infants may develop allergic reactions, with studies indicating that approximately 2.5% of infants may develop a cow’s milk allergy during their first year of life. Investigations into this clearly focus on the different protein content of cow’s milk compared to human breast milk [9,10]. Unfortunately, once an allergic reaction occurs, it is often difficult to treat. The most common approach is to eliminate certain foods that trigger the allergy, but this method can increase the risk of malnutrition and negatively impact the infant’s growth.

It has been reported that the composition of milk varies during the lactation period, and describing milk using a single composition is invalid [1,11,12]. For example, the protein content in milk can vary in each phase depending on the type of mammal and the needs of the individual newborn. Various proteins contribute to milk’s dynamic quality, and these could be used as indicators when managing the feeding of newborns. Over the last few years, widespread interest in the nutritional quality of milk and its association with infant health care has resulted in scientific research on the composition of various milk types [13].

A growing demand has emerged for donkey’s milk from various consumers due to its proven high digestibility; antiviral, antimicrobial, antioxidant, and anti-inflammatory properties; and relevance for patients suffering from protein allergies [14,15,16,17]. Due to its health-promoting effects, described by many authors, donkey milk could be a functional food [18]. Furthermore, scientific evidence has indicated the similarity between donkey and human milk compositions [13,19]. However, changes in the composition of donkey milk throughout the lactation period have been poorly investigated. Only a few studies have studied the lactation period for longer than five or six months, and the fully productive potential (9 to 10 months) has not been assessed effectively [18,20,21].

The aim of our study was to investigate bioactive protein content alterations in human and donkey milk from the first to the ninth month of lactation, using the electrophoresis method to analyze similarities in their protein content, and covering the full lactation period of the tested asses.

## 2. Materials and Methods

### 2.1. Human Milk Sample Collection

The collection of human breast milk was approved by the Ethics Committee of the Medical Faculty of Trakia University, Bulgaria (protocol № 33/09.09.2024). Thirteen women aged 21 to 40 were voluntarily included in the study. All donors were individually consulted and acquainted with the objectives of the investigation, and informed consent was obtained from each of them. The breast milk samples (a total of 63) were collected after the daily needs of infants were satisfied by pumping immediately after each suckle according to the scheme listed below. This process additionally stimulates lactation to produce enough breast milk to feed the infant. The samples were obtained from clinically healthy mothers who did not use additional medications to stimulate lactation, did not smoke, or consume alcohol; the sample collection procedure described in the information leaflet was strictly followed. All samples were assembled from the mothers of full-term babies in the lactation stage from 10 to 273 days (9 months). The breastfeeding periods occurred no less than six times per day during the first five months but decreased as lactation progressed.

Briefly, 250 to 300 mL of human milk was extracted per day by the women using sterile automatic breast pumps. The first few drops were discarded, and the collected milk samples were immediately shipped to the laboratory on dry ice or in a refrigerator at −18 °C, where they were kept no longer than one week. In the laboratory, all samples were stored at −80 °C before the start of the experiment. Directly before the investigation, monthly samples were prepared by mixing an equal quantity of the seven randomly chosen donated human breast milk samples from each of the studied months, which were classified into one group. Consequently, nine representative samples were assembled, one for each of the nine lactation months, strictly following general milk sample procedures.

### 2.2. Donkey Milk Sample Collection

**Scheme 1:**
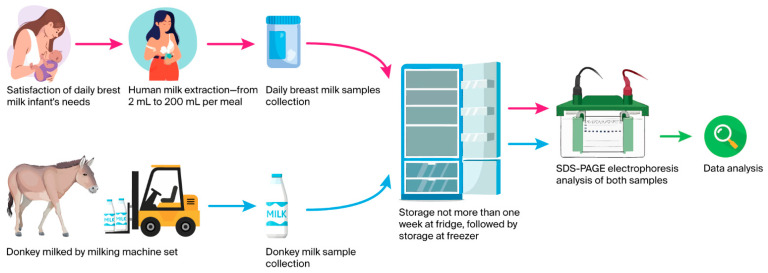
Graphic explanation of the experimental design.

Donkey milk samples were taken from 60 healthy local Bulgarian donkeys, which were selected from 540 animals, during the lactation period of 273 days (9 months). The total group of nine donkey milk samples was formed by mixing collected milk samples and strictly following general milk sample procedures. Thus, all lactation stages of the asses were studied, covering the colostrum period, transitional milk, and late lactation phase. The milk samples were collected during morning mechanical milking (vacuum pressure 42 kPa; pulsation ratio, 50:50; pulsation rate, 120 cycles/min), cooled to −18 °C, and stored at −80 °C until electrophoresis analysis was performed (Scheme 1). The donkeys were raised in a semi-closed house on a farm with an animal husbandry capacity of up to 540 asses located at the Sredna Gora area, near Yambol city, Bulgaria. All donkeys drank freely and were offered the same diet of grass hay, supplemented with grain feed.

### 2.3. Sodium Dodecyl Sulfate Polyacrylamide Gel Electrophoresis of Protein Fractions

Sodium dodecyl sulphate polyacrylamide gel electrophoresis (SDS-PAGE) was performed as described by Laemmli (1970) [22] under reducing conditions, using the OmniPAGE WAVE Maxi Electrophoresis System, Cleaver Scientific, Warwickshire, UK (gel size 20 cm × 20 cm) and concentration ratio of acrylamide and bis-acrylamide at 6% and 10%, respectively (Bio-Rad Laboratories, Inc., Hercules, CA, USA). α-casein, β-casein, and ɑ-lactalbumin from bovine milk; β-lactoglobulin from bovine milk; bovine serum albumin (Sigma-Aldrich, Darmstadt, Germany); and protein test mixture 6 (SERVA Electrophoresis GmbH, Heidelberg, Germany) containing phosphorylase B (97.4 kDa), ovalbumin (45 kDa), carbonic anhydrase (29 kDa), soybean trypsin inhibitor (21 kDa), cytochrome C (12.5 kDa), and aprotinin (6.5 kDa) were used for SDS analysis. All samples were prepared following the procedure described below. Defrosted milk samples were dissolved in a 1:1 ratio with Tris-HCl buffer (0.1 M pH 7.0). The 500 µL samples were mixed with 500 µL Tris-HCl sample buffer (0.1 M Tris-HCl buffer, pH 6.8, 2.0% SDS, 50 mM dithiothreitol, 10% glycerol, 0.002% bromphenol blue) and heated at 90 °C for 3 min before being loaded onto the gel. Electrophoresis was performed at room temperature with a constant current of 30 mA. The Rf (retardation factor) value was determined from the expressed protein fractions in the samples, and their molecular weights were estimated using GelAnalyzed-19.1 software, where standard molecular weights of the common proteins were used.

### 2.4. Statistical Analysis

This study’s data were statistically analyzed by IBM SPSS Statistics 27.0 using descriptive statistics, Pearson’s correlation, the chi-square test, and Student’s *t*-test. Categorical data were organized into 2 × 2 contingency tables. A *p* value below 0.05 was labeled statistically significant. The results are presented in both diagram (created using Excel on Windows 10) and table formats.

## 3. Results and Discussion

**Figure 1:**
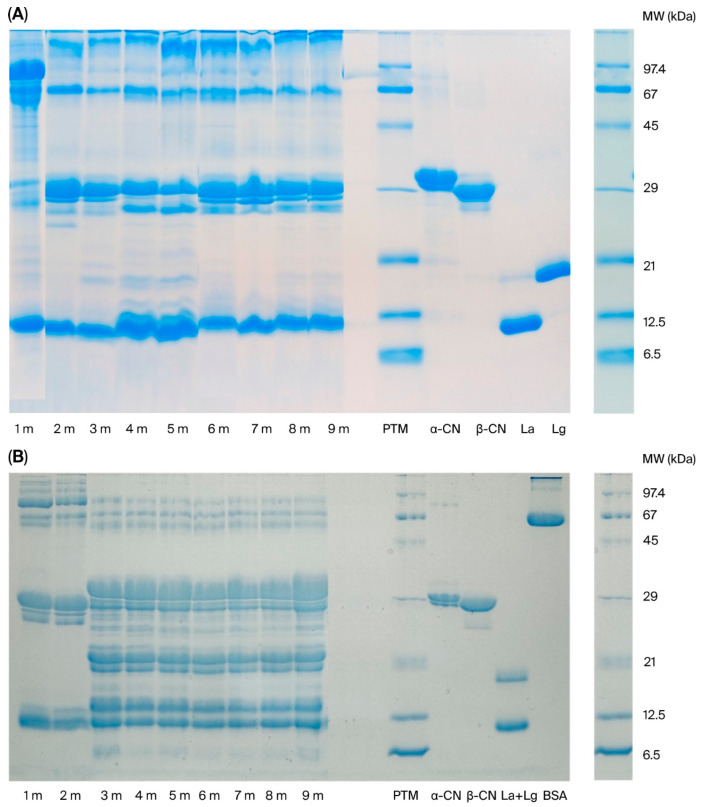
The imaging obtained via the SDS polyacrylamide gel electrophoresis analysis of the studied milk samples, which are listed as follows: (**A**) human breast milk and (**B**) donkey milk. Numbers 1 to 9 indicate the different months in which the milk samples were collected. The definitions of abbreviations are provided as follows: PTM—protein test mixture; α-CN—α-casein fraction; β-CN—β-casein fraction; La—lactalbumin; Lg—lactoglobulin; BSA—serum albumin.

Images of the proteins identified in human and donkey milk samples, obtained via SDS polyacrylamide gel electrophoresis analysis, are presented in Figure 1A,B. SDS polyacrylamide gel electrophoresis (SDS-PAGE) enabled the analysis of protein complex structures by promoting their dissociation in detergent sodium dodecyl sulfate (SDS). Under an applied voltage gradient, the protein structures were separated into individual polypeptide chains according to their charges and molecular weights. They were observed as migrating narrow bands or discs through the gel and then identified based on the migration distances of the dye front and calculating Rf (retardation factor) values according to the Rf values of the marker proteins (the protein test mixture).

Based on the GelAnalyzer-19.1 software, the molecular weights were calculated for the proteins identified in the tested human and donkey milk samples. The data are presented in Table 1 and Table 2.

**Figure 2:**
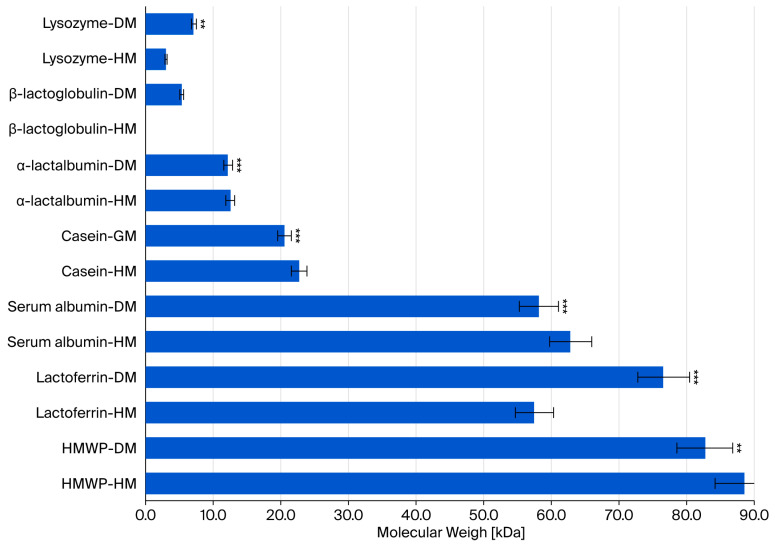
Comparative analysis between proteins detected in human and donkey milk samples. Levels of statistical significance: ** *p* < 0.01 and *** *p* < 0.001 between individual proteins in human and donkey milk samples. Abbreviations: DM—donkey milk; HM—human milk; HMWP—high-molecular-weight protein.

All samples collected during the first to ninth months of lactation from humans and donkeys covered the entire lactation period. A comparative analysis was performed on the average molecular weight values calculated for proteins obtained from human and donkey milk, the results of which are displayed in Figure 2. Statistically significant results were expected for each protein examined in both samples. The correlation coefficients are shown in Table 3.

The results identified similarities in the protein fractions for both the donkey and human milk samples tested. The data analysis showed that casein, as well as whey proteins, such as lactoferrin, serum albumin, α-lactalbumin, lysozyme, and low-molecular-weight peptides, were detected simultaneously in the human and donkey milk samples using the SDS-PAGE method.

The results for human and donkey milk casein fractions correlate with those of previously reported SDS-PAGE studies [23,24,25]. During human milk testing, a deep line appeared corresponding to the β-casein fraction (from 25 to 27 kDa), which was expressed continually from the second to the ninth month (Figure 1A). It is well known that breast milk is characterized by the presence of the highly phosphorylated protein β-casein, which contributes to the high bioavailability of calcium in the milk and improves the absorption of other divalent cations, such as iron and zinc. It has a beneficial effect on newborn health [26,27]. A distinct result was observed in the probe obtained from the first month (Table 1). The intensified colostrum period and consequent secretion of transitional milk resulted in higher levels of whey proteins, such as lactoferrin (84 kDa), serum albumin (65.1 kDa), α-lactoalbumine (13.4 kDa), and lysozyme (16.1 kDa), for the first month of human milk samples. It is widely known that colostrum and transitional milk contain an assembly of unique components, including macronutrients, from which whey proteins, micronutrients, antimicrobials, and growth factors emerge. These components stimulate the immune system and healthy growth of newborns [28]. The lack of hydrochloric acid in newborn stomachs, which is connected with activation of pepsin from pepsinogen, makes casein protein digestion and its absorption more difficult than that of whey proteins [29]. For this reason, milk secretion at the onset of lactation, the period covering colostrum and transitional milk, has important effects on newborn health, such as providing whey proteins. These contribute to the function of the immune and gastrointestinal development [30].

According to the SDS-PAGE results obtained, the donkey milk samples collected during the third to ninth months possess a similar electrophoretic profile (Figure 1B, Table 2). The four types of casein fractions were identified, from which α- and β-casein were more highly expressed based on the estimation of their molecular weights (approximately 27 kDa for α-casein and 25 kDa for β-casein). The percentage ratio of β-casein to α-casein in the casein fractions was calculated using the GelAnalyzer program at 49.5 ± 3.0% and 31.5 ± 3.0%, respectively. This data confirms the similarity between donkey and human milk (Figure 2), regarding the β-casein fraction being the prevailing fraction in both milk types [14]. The other lighter fractions, which most likely correspond to αS2-casein and κ-casein, were found to have a minor content in donkey milk. These results for donkey’s milk correlate with previously reported studies [14,31,32,33].

It was interesting to note that the first and second donkey milk samples tested displayed different electrophoretic patterns (Figure 1B, Table 2); an increase in intensity of bands associated with high-molecular-weight whey proteins was detected (Mw from 99.2 to 117.5 kDa). Additionally, no β-casein (25 kDa) and α-casein (27 kDa) fractions or β-lactoglobulin (17–18 kDa) were detected during this period (Table 2). One possible explanation is the extended transition milk phase in the tested donkey milk, which differs from human milk. Stable levels of high-molecular-weight whey protein were identified during all tested periods (from the 1st to 9th months of lactation), similar to human milk (Figure 2). Our results have confirmed that donkey milk is particularly rich in whey proteins [34] and contains low amounts of casein, where β-casein is the predominant fraction [14,31,32,33]. These characteristics of donkey milk are distinct from ruminant milk but make it closer in composition to human milk. Moreover, the longer transition milk lactation period registered in the tested donkey milk samples could play an important physiological role. Because of its high digestibility and prominent whey protein content compared to casein fractions, it contributes to the development of immune and gastrointestinal regulation, while preventing milk allergies [35,36]. In newborns where a limited digestive function is observed during the first few months of life and in infants diagnosed with protein allergies, donkey milk could be used as an alternative “pharmafood”.

Based on a literary examination and comparative molecular weight analysis of the data obtained, we also detected lactoferrin expression (approximately from 71 kDa to 84 kDa) across the entire lactation period in both donkey and human milk samples (Table 1 and Table 2; Figure 2). The variations noted in the molecular weight of lactoferrin in both samples studied can be explained by the period of lactation, where higher-molecular-weight lactoferrin was identified during the intensive colostrum period. According to Czosnykowska-Łukacka et al.’s study [37], the concentration of lactoferrin in human milk varies with the lactation stage, but the highest concentration is observed during the colostrum period. There is evidence that the higher concentration of lactoferrin during early infancy promotes the proliferation of intestinal epithelial cells, and when lactoferrin concentration decreases, cell differentiation is stimulated [38]. In donkey milk, the quantity of lactoferrin, estimated as a percentage of the total whey protein, was 4.48%, which is between that of cow and human milk [39].

The 692–697 amino acids forming the polypeptide chain of lactoferrin significantly contribute to supplying breast-feeding infants with a mixture of amino acids. In addition to its well-known antimicrobial activity expressed by its iron-chelating ability [40,41], lactoferrin possesses anti-inflammatory and immunomodulating activity [42]. There is evidence that lactoferrin acts as a mucosal immune response activator by upregulating secretory IgA production. Its main function is to maintain microbial homeostasis in the gut and increase the resilience of probiotic populations [43]. In donkey milk, the immunostimulant activity of lactoferrin is confirmed due to its ability to bind to Toll-like receptor 4 (TLR4) on monocytes and macrophages, triggering the NK-κB signaling pathway, which upregulates the expression of pro-inflammatory cytokines (IL-1β, IL-6, TNF-α) during infections [44,45]. Another study by Likaa et al. [46] showed that the oral administration of donkey milk lactoferrin in mice infected with Serratia liquefaciens (experimental design) reduced the number of bacteria in the lung and decreased levels of tumor necrosis factor-alpha (TNF-α), IL-6, and other cytokines. It minimized the inflammatory response in mice lung tissues induced by Serratia liquefaciens while activating the innate immune response [46].

Similar to the lactoferrin protein, serum albumin (molecular weight 66 ± 2 kDa) was observed in both human and donkey milk throughout the entire lactation period studied (Table 1 and Table 2; Figure 2). The results indicate that during the first month, serum albumin levels in human milk were significantly higher than in the rest of the months. Compared to human milk, serum albumin in donkey milk samples remained high during the first and second months. It is widely accepted that the protein content may vary depending on environmental factors such as diet, but it is also affected by the lactation phase [8]. Our results confirm the findings of previous studies, in which the levels of serum albumin were studied in breast milk over 6 months, with the data indicating higher serum albumin contents during the first few months [47,48]. The distinction between serum albumin and other milk whey proteins is their secretion source. Most proteins are synthesized by the mammary epithelium, but serum albumin is obtained directly via the mother’s blood circulation [1]. It contributes to the maintenance of colloid osmotic pressure, supplying infants with essential amino acids, and transports various substances through the circulation [49,50]. Moreover, serum albumin maintains the infant’s immune system before they are able to form their own immune cells [47].

According to our analysis of lysozyme, this enzyme was detected predominantly in the third to ninth months of lactation in donkey milk samples (Figure 2, Table 2). We also detected lysozyme in human breast milk samples, but not to the extent seen in donkey milk (Figure 2). These results are supported by previous observations for lysozyme content in donkey and human milk [13,51,52]. The enzyme is part of an innate immune response and possesses an antimicrobial function that reduces the incidence of gastrointestinal infections [53] and exerts a selective action on gut bacteria [54]. In infants, lysozyme inhibits the spread of bacterial pathogens [13,55].

Human milk is the first and best option for infant feeding, and, to date, no adverse effects have been found related to its intake. Our investigation demonstrated a similarity between the contents of caseins and other proteins in the tested donkey and human milk samples during the entire lactation period. This highlights donkey milk as a possible adequate alternative to human milk when allergen responses are detected in response to the proteins present in cow’s milk.

## 4. Conclusions

Our study is the first to analyze protein contents in human and donkey milk samples collected across the entire lactation period (nine months). Comparable protein contents were found in human and donkey milk, which correlates with reports from previous short-term observations made by other studies. The similarities between human and donkey milk proteins could lead to more clinical studies in which donkey milk is used in milk-based infant formulas.

## Figures and Tables

**Table 1:** Molecular weight values calculated for proteins detected in human breast milk samples donated during location from months 1 to 9.

Molecular Weight of Proteins from Human Breast Milk Samples								
1 Month	2 Months	3 Months	4 Months	5 Months	6 Months	7 Months	8 Months	9 Months
123.5	123.3	95.0	125.9	92.2	126.2	130.6	125.8	89.7
83.6	84.1	80.5	83.1	68.3	114.9	82.5	91.8	69.5
69.4	72.1	68.4	68.3	27.4	85.7	65.9	81.9	27.7
27.4	65.1	27.7	26.8	26.8	69.5	27.1	66.3	26.1
23.9	54.6	24.4	25.4	24.5	26.6	26.7	50.1	24.5
18.2	49.2	16.1	23.7	17.1	23.5	23.3	26.5	16.2
16.0	28.9	14.6	16.5	13.7	21.4	17.0	24.2	13.7
14.3	26.7	13.5	13.6		16.4	15.1	16.3	
13.4	24.7				13.4	13.3	13.7	
	17.2							
	16.1							
	13.4							

The colors indicate different established protein fractions as follows: yellow—high-molecular-weight whey proteins; blue—lactoferrin; purple—serum albumin; grey—caseins; green—α-lactalbumin; orange—lysozyme; colorless—low-molecular-weight peptides.

**Table 2:** Molecular weight values calculated for proteins detected in donkey milk samples during lactation from months 1 to 9.

Molecular Weight of Proteins in Donkey Milk Samples								
1 Month	2 Months	3 Months	4 Months	5 Months	6 Months	7 Months	8 Months	9 Months
113.9	117.5	91.0	87.5	85.2	83.3	85.7	87.7	89.7
99.2	104.8	79.8	79.3	71.4	71.5	71.5	72.7	74.8
82.0	82.9	63.0	63.3	64.5	64.0	65.5	66.0	64.2
67.6	69.0	28.0	27.3	27.3	27.0	26.8	28.0	28.5
61.2	64.7	25.6	25.3	24.6	25.0	24.3	25.2	25.2
24.5	24.9	22.9	24.1	23.4	23.7	23.8	24.0	23.9
23.1	23.0	22.1	22.8	22.1	22.2	22.5	22.5	20.4
21.7	21.4	20.8	20.4	20.1	20.6	20.0	20.4	19.6
13.9	13.9	18.1	19.6	19.1	16.6	16.7	16.7	18.1
13.7	13.5	16.7	18.2	17.7	15.9	16.0	16.0	16.8
		16.0	16.8	16.5	14.0	13.9	14.0	16.1
		14.9	16.1	15.8	13.4	13.4	13.4	15.0
		14.0	14.0	13.9				13.9
		13.4	13.5	13.4				13.4

The colors indicate different established protein fractions as follows: yellow—high-molecular-weight whey proteins; blue—lactoferrin; purple—serum albumin; grey—caseins; brown—β-lactoglobulin; green—α-lactalbumin; orange—lysozyme; colorless—low-molecular-weight peptides.

**Table 3:** The correlation coefficients between individual proteins detected in human and donkey milk samples.

	Lysozyme	Lactalbumin	Caseins	Serum Albumin	Lactoferrin	HMWP
Pearson’s correlation coefficient	0.129	0.160	<0.001	0.144	0.144	0.014

## Data Availability

The original contributions presented in this study are included in the article. Further inquiries can be directed to the corresponding author.
